# Contribution of an Asian-prevalent HLA haplotype to the risk of HBV-related hepatocellular carcinoma

**DOI:** 10.1038/s41598-023-40000-3

**Published:** 2023-08-09

**Authors:** Atsushi Kawamura, Koichi Matsuda, Yoshinori Murakami, Masayuki Saruta, Takashi Kohno, Kouya Shiraishi

**Affiliations:** 1 Division of Genome Biology, National Cancer Center Research Institute, 5-1-1, Tsukiji, Chuo-ku, Tokyo, 104-0045 Japan; 2 Laboratory of Clinical Genome Sequencing, Department of Computational Biology and Medical Sciences, Graduate School of Frontier Sciences, The University of Tokyo, 4-6-1 Shirokanedai, Minato-ku, Tokyo, 108-8639 Japan; 3 Division of Molecular Pathology, Department of Cancer Biology, Institute of Medical Science, University of Tokyo, 4-6-1, Shirokanedai, Minato-ku, Tokyo, 108-8639 Japan; 4 Division of Gastroenterology and Hepatology, Department of Internal Medicine, The Jikei University School of Medicine, 3-25-8, Nishi-Shimbashi, Minato-ku, Tokyo, 105-8461 Japan

**Keywords:** Cancer, Cancer genomics

## Abstract

Liver cancer, particularly hepatitis B virus (HBV)-related hepatocellular carcinoma (HCC), is more common in Asians than in Caucasians. This is due, at least in part, to regional differences in the prevalence of exogenous factors such as HBV; however, endogenous factors specific to Asia might also play a role. Such endogenous factors include HLA (human leukocyte antigen) genes, which are considered candidates due to their high racial diversity. Here, we performed a pancancer association analysis of 147 alleles of HLA-class I/II genes (HLA-A, B, and C/DRB1, DQA1, DQB1, DPA1, and DPB1) in 31,727 cases of 12 cancer types, including 1684 liver cancer cases and 107,103 controls. HLA alleles comprising a haplotype prevalent in Asia were significantly associated with pancancer risk (e.g., odds ratio [OR] for a DRB1*15:02 allele = 1.12, *P* = 2.7 × 10^–15^), and the associations were particularly strong in HBV-related HCC (OR 1.95, *P* = 2.8 × 10^–5^). In silico prediction suggested that the DRB1*15:02 molecule encoded by the haplotype does not bind efficiently to HBV-derived peptides. RNA sequencing indicated that HBV-related HCC in carriers of the haplotype shows low infiltration by NK cells. These results indicate that the Asian-prevalent HLA haplotype increases the risk of HBV-related liver cancer risk by attenuating immune activity against HBV infection, and by reducing NK cell infiltration into the tumor.

## Introduction

Cancer incidence differs according to ethnicity^1^. Specifically, hepatocellular carcinoma (HCC) linked to hepatitis B virus (HBV) infection occurs more frequently among Asians than Caucasians^2^. HCC comprises 80% of all hepatic malignancies worldwide^3^. HBV or hepatitis C virus (HCV) infection is the primary etiology underlying persistent hepatic disease and the consequent emergence of HCC; however, there is a significant subset of HCC cases that are negative for both HBV and HCV infection, referred to as non-B non-C HCC (NBNC HCC)^4^. Differences in the prevalence of HBV across various regions account, at least in part, for the difference in incidence of HBV-related HCC; however, the increased incidence of HCC among Japanese Americans highlights the potential contribution of endogenous factors that are shared by Asians^5^. Polymorphisms in genes encoding human leukocyte antigens (HLAs) are strong candidates as endogenous determinants given that the distribution of these polymorphic alleles varies substantially across ethnic groups^6^. Neoantigens arising from somatic mutations in tumor cells are presented as peptides by HLA-class I molecules on tumor cells and are recognized by CD8+ (cytotoxic) T cells, which trigger killing of tumor cells. Moreover, HLA-class II molecules expressed on immune cells play a critical role in antitumor immune responses by binding to viral antigens^7^. Hence, it is plausible that interethnic disparities in the distribution of polymorphic HLA alleles may underpin interethnic discrepancies in susceptibility to cancer by modifying the anticancer immune response.

HLA genes, specifically HLA-A, -B, and -C (class I) and HLA-DRB1, DQA1, DQB1, DPA1, and DPB1 (class II), are highly polymorphic. These polymorphisms exist in linkage disequilibrium within the major histocompatibility complex (MHC) region, resulting in extended haplotypes^8^. The frequencies of these haplotypes, as well as alleles of the eight HLA genes, differ according to ethnicity. For example, A*24:02-B*52:01-C*12:02-DRB1*15:02-DQA1*01:03-DQB1*06:01-DPA1*02:01-DPB1*09:01 is the most common haplotype in Japanese individuals (8.30%)^8^, while it is rare in populations of European ancestry (0.34%)^9^. Hence, it is highly plausible that HLA alleles/haplotypes may account for the heightened susceptibility to various cancer types observed in Asian populations. However, to the best of our knowledge, no comprehensive large-scale study has been conducted to investigate the association between HLA alleles and cancer risk in Asian populations. This is due, at least partly, to the challenge of accurately imputing HLA alleles from genome-wide single nucleotide polymorphism (SNP) data, which has limited the inclusion of HLA alleles in genome-wide association studies (GWASs)^10–17^.

Here, we conducted a large-scale pancancer association analysis of HLA-A, -B, and -C (class I) and HLA-DRB1, DQA1, DQB1, DPA1, and DPB1 (class II) genes to address whether specific HLA alleles contribute to cancer risk. The study subjects included 31,727 cancer cases (12 cancer types in total) and 107,103 noncancer controls; their genome-wide SNP typing data were available in the BioBank Japan database (https://biobankjp.org/), and all were genetically matched with each other for the study. The availability of the Japanese HLA reference panel enabled us to perform precise imputation of HLA alleles from the SNP data^18^. The outcomes of the association analysis demonstrate that HLA alleles forming a prevalent haplotype in Asian populations exhibit a substantial correlation with the overall risk of pancancer, notably displaying a markedly strong association with the risk for HBV-related HCC. Plausible molecular mechanisms underlying the association between HLA alleles and HCC risk were inferred through in silico analysis of the binding of HLA-DRB1 to HBV-derived peptides, as well as the mutational and transcriptional profiles of 160 HCC tissue samples collected from Japanese patients.

## Results

### Pancancer association analysis of HLA alleles

Subjects were selected carefully based on their genetic background (see “Materials and Methods”). The study examined 31,727 cases of cancer (12 different types) and 107,103 controls. The 12 types were colon cancer (n = 6,854), stomach cancer (n = 6,424), breast cancer (n = 5,476), prostate cancer (n = 5,311), lung cancer (n = 3,919), liver cancer (n = 1,684), esophageal cancer (n = 1,274), uterine cancer (n = 975), ovarian cancer (n = 704), cervical cancer (n = 523), pancreatic cancer (n = 408), and gallbladder/bile duct cancer (n = 318). Controls comprised individuals diagnosed with noncancerous diseases (Supplementary Table 1). The MHC region was comprehensively imputed with a Japanese HLA reference panel^8^, thereby enabling analysis of 147 alleles in total: 17 HLA-A alleles, 33 HLA-B alleles, and 18 HLA-C alleles, as well as 26 HLA-DRB1 alleles, 18 HLA-DQA1 alleles, 15 HLA-DQB1 alleles, four HLA-DPA1 alleles, and 16 HLA-DPB1 alleles. Association analysis was performed for 68 HLA-class I and 79 class II alleles.

After Bonferroni correction, we found a significant association between pancancer risk and 12 alleles (*P* < 0.00034 [= 0.05/147]). Notably, eight of these 12 alleles (A*24:02, B*52:01, C*12:02, DRB1*15:02, DQA1*01:03, DQB1*06:01, DPA1*02:01, and DPB1*09:01) form a haplotype that is the most prevalent in Japanese populations (Supplementary Table 2), but rare in Caucasian populations^8^. The eight alleles were commonly linked to an increased cancer risk (Table 1**,** OR of a DRB1*15:02 allele = 1.12, *P* = 2.7 × 10^–15^). In this article, we refer to this haplotype as the "Asian-prevalent HLA haplotype".

**Table 1:** Association of HLA alleles with pancancer risk.

HLA gene	Allele	Case	Control	OR^a^	95% CI		*P* value
		Allele frequency			Lower	Upper	
HLA-A	24:02^b^	0.380	0.369	1.05	1.03	1.07	5.2E−07
HLA-B	52:01^b^	0.124	0.114	1.10	1.07	1.13	2.6E−12
HLA-B	40:02	0.073	0.078	0.94	0.91	0.97	1.1E−04
HLA-C	12:02^b^	0.124	0.114	1.10	1.07	1.13	1.2E−12
HLA-DRB1	15:02^b^	0.116	0.105	1.12	1.09	1.15	2.7E−15
HLA-DRB1	08:02	0.040	0.043	0.92	0.88	0.96	3.0E−04
HLA-DQA1	01:03^b^	0.201	0.187	1.10	1.07	1.12	1.7E−15
HLA-DQA1	01:02	0.137	0.143	0.95	0.93	0.98	1.2E−04
HLA-DQB1	06:01^b^	0.196	0.182	1.09	1.07	1.12	4.8E−15
HLA-DPA1	02:01^b^	0.159	0.151	1.07	1.04	1.09	2.4E−07
HLA-DPA1	01:03	0.392	0.400	0.97	0.95	0.98	1.5E−04
HLA-DPB1	09:01^b^	0.105	0.096	1.10	1.07	1.13	1.4E−10

^a^Adjusted for age, sex, and the top five major PCA components, which were obtained from the pancancer GWAS.

^b^Alleles constituting an Asian-prevalent haplotype.

HLA: human leukocyte antigen, OR: odds ratio, CI: confidence interval.

Subsequently, we investigated the association between the eight alleles comprising the Asian-prevalent haplotype and the risk for each of the 12 cancer types. After applying Bonferroni correction, we identified significant associations with four cancer types: liver, stomach, cervical, and lung (Table 2**;** e.g., the OR of a DRB1*15:02 allele for liver cancer risk = 1.30, *P* = 3.1 × 10^–7^). Notably, three of these cancers (liver, stomach, and cervical) are more prevalent in Asian populations than in Caucasian populations, and are linked to viral or bacterial infections^1^. In light of the availability of information regarding cancer subtypes and viral infection, we focused on liver cancer in the subsequent part of this study.

**Table 2:** Association of HLA alleles comprising an Asian-prevalent haplotype with cancer risk.

Type (N)	Class I			Class II				
	A*24:02	B*52:01	C*12:02	DRB1*15:02	DQA1*01:03	DQB1*06:01	DPA1*02:01	DPB1*09:01
	OR^a^ (95% CI) *P* value	OR^a^ (95% CI) *P* value	OR^a^ (95% CI) *P* value	OR^a^ (95% CI) *P* value	OR^a^ (95% CI) *P* value	OR^a^ (95% CI) *P* value	OR^a^ (95% CI) *P* value	OR^a^ (95% CI) *P* value
Pancancer^b^ (31,727)	**1.05** **(1.03–1.07)** **5.2E−07**	**1.10** **(1.07–1.13)** **2.6E−12**	**1.10** **(1.07–1.13)** **1.2E−12**	**1.12** **(1.09–1.15)** **2.7E−15**	**1.10** **(1.07–1.12)** **1.7E−15**	**1.09** **(1.07–1.12)** **4.8E−15**	**1.07** **(1.04–1.09)** **2.4E−07**	**1.10** **(1.07–1.13)** **1.4E−10**
Liver cancer (1,684)	1.10 (1.03**–**1.18) 7.0E−03	**1.26** **(1.14–1.39)** **5.8**E−**06**	**1.25** **(1.14–1.38)** **7.9E−06**	**1.30** **(1.18–1.44)** **3.1E−07**	**1.24** **(1.15–1.35)** **1.7E−07**	**1.27** **(1.17–1.38)** **1.3E−08**	1.17 (1.07**–**1.28) 6.1E−04	**1.26** **(1.13–1.40)** **2.3E−05**
Stomach cancer (6,424)	1.07 (1.03**–**1.11) 7.0E−04	**1.13** **(1.07–1.20)** **5.1E−06**	**1.14** **(1.08–1.20)** **3.1E−06**	**1.16** **(1.10–1.23)** **1.1E−07**	**1.14** **(1.09–1.19)** **4.5E−09**	**1.14** **(1.09–1.20)** **9.4E−09**	1.08 (1.02**–**1.13) 3.4E−03	**1.11** **(1.05–1.18)** **2.9E−04**
Cervical cancer (523)	1.21 (1.07**–**1.37) 2.1E−03	**1.51** **(1.28–1.78)** **1.4E−06**	**1.51** **(1.28–1.78)** **1.2E−06**	**1.47** **(1.24–1.75)** **1.3E−05**	**1.34** **(1.16–1.55)** **5.4E−05**	**1.34** **(1.16–1.55)** **6.3E−05**	1.29 (1.11**–**1.51) 1.2E−03	**1.43** **(1.20–1.72)** **9.3E−05**
Lung cancer (3,919)	1.07 (1.02**–**1.12) 4.8E−03	**1.17** **(1.09–1.25)** **5.7E−06**	**1.17** **(1.09–1.25)** **5.9E−06**	**1.16** **(1.08–1.24)** **3.6E−05**	**1.19** **(1.12–1.25)** **1.3E−09**	**1.19** **(1.13–1.26)** **5.6E−10**	1.07 (1.00**–**1.14) 3.8E−02	1.12 (1.04**–**1.21) 2.4E−03
Breast cancer (5,476)	1.04 (1.00**–**1.09) 5.5E−02	1.08 (1.02**–**1.15) 1.2E−02	1.09 (1.02**–**1.16) 7.2E−03	1.10 (1.03**–**1.17) 4.8E−03	1.07 (1.02**–**1.12) 1.1E−02	1.06 (1.01**–**1.12) 1.6E−02	1.05 (0.99**–**1.11) 1.0E−01	1.07 (1.01**–**1.15) 3.6E−02
Colon cancer (6,854)	1.04 (1.00**–**1.08) 3.7E−02	1.05 (0.99**–**1.10) 9.4E−02	1.04 (0.99**–**1.10) 1.2E−01	1.06 (1.00**–**1.12) 4.1E−02	1.05 (1.00**–**1.09) 3.7E−02	1.04 (1.00**–**1.09) 6.7E−02	1.06 (1.01**–**1.11) 1.9E−02	1.06 (1.00**–**1.12) 5.8E−02
Prostate cancer (5,311)	1.04 (1.00**–**1.08) 8.1E−02	1.06 (1.00**–**1.13) 6.7E–02	1.06 (1.00**–**1.13) 5.4E−02	1.08 (1.02**–**1.15) 1.3E−02	1.02 (0.97**–**1.08) 3.8E−01	1.02 (0.97**–**1.08) 3.6E−01	1.03 (0.98**–**1.06) 2.6E−01	1.08 (1.01**–**1.15) 2.0E−02
Gallbladder & bile duct cancer (318)	1.02 (0.87**–**1.20) 8.1E−01	1.24 (0.99**–**1.55) 6.2E−02	1.26 (1.01**–**1.58) 4.4E−02	1.25 (0.99**–**1.58) 5.7E−02	1.28 (1.07**–**1.54) 8.0E−03	1.29 (1.07**–**1.55) 7.2E−03	1.09 (0.88**–**1.34) 4.5E−01	1.32 (1.04**–**1.67) 2.1E−02
Esophageal cancer (1,274)	1.01 (0.93**–**1.10) 7.5E−01	1.02 (0.90**–**1.15) 7.8E−01	1.02 (0.90**–**1.15) 8.1E−01	1.01 (0.89**–**1.14) 9.2E−01	1.03 (0.93**–**1.14) 5.7E−01	1.03 (0.93**–**1.14) 5.5E−01	1.05 (0.94**–**1.17) 4.0E−01	1.04 (0.91**–**1.18) 5.7E−01
Pancreatic cancer (408)	0.92 (0.80**–**1.06) 2.5E−01	1.00 (0.80**–**1.24) 9.9E−01	1.01 (0.82**–**1.26) 9.1E−01	1.00 (0.80**–**1.25) 9.9E−01	0.97 (0.81**–**1.16) 7.2E−01	0.96 (0.80**–**1.15) 6.4E−01	1.11 (0.92**–**1.33) 2.9E−01	0.89 (0.70**–**1.14) 3.6E−01
Uterine cancer (975)	1.09 (0.99**–**1.19) 7.9E−02	1.12 (0.98**–**1.28) 1.1E−01	1.13 (0.99**–**1.29) 7.5E−02	1.13 (0.98**–**1.30) 8.7E−02	1.08 (0.97**–**1.21) 1.5E−01	1.07 (0.95**–**1.19) 2.7E−01	1.10 (0.97**–**1.24) 1.4E−01	1.12 (0.97**–**1.29) 1.3E−01
Ovarian cancer (704)	1.01 (0.91**–**1.13) 8.6E−01	1.21 (1.03**–**1.41) 1.7E−02	1.22 (1.05**–**1.43) 1.0E−02	1.27 (1.08**–**1.48) 3.0E−03	1.14 (1.00**–**1.30) 5.0E−02	1.13 (1.00**–**1.29) 5.8E−02	1.07 (0.92**–**1.23) 3.8E−01	1.17 (0.99**–**1.38) 6.7E−02

^a^Adjusted for age, sex, and the top five major PCA components, which were obtained from the pancancer GWAS.

Results with statistically significant associations are shown in bold.

OR: odds ratio; CI: confidence interval.

^b^Includes 2143 multiple primary cancers.

### Liver cancer association analysis of HLA alleles

HBV and HCV infections are the primary etiologies of liver cancer^2,3^. Hence, we investigated the differences in the association of HLA alleles in the context of viral infection. Our analysis encompassed cases of HBV-related liver cancer/HCC (n = 128/67), HCV-related liver cancer/HCC (n = 622/299), and virus-negative liver cancer/HCC (NBNC) (n = 277/130). Notably, we found that the ORs for the associations across seven HLA genes, except for HLA-A, were significantly higher for HBV-related cancers than for HCV-related and NBNC cancers (Table 3**;** e.g., the OR of a DRB1*15:02 allele for HBV-related liver cancer = 1.95, *P* = 2.8 × 10^–5^). Furthermore, when we limited our analysis to cases with information related to the diagnosis of carcinoma (i.e., HCC), we detected an even more substantial increase in the ORs (Table 3; e.g., the OR of a DRB1*15:02 allele for HBV-related HCC = 2.43, *P* = 1.7 × 10^–5^). Our case-case analysis comparing HBV-related cancer with NBNC cancers (i.e., NBNC cases were used as a reference) also demonstrated significant differences, suggesting that HLA alleles comprising the Asian-prevalent haplotype exhibit a stronger association with the risk for HBV-related liver cancer than for NBNC liver cancer (Supplementary Table 3).

**Table 3:** Association analysis of HLA alleles with liver cancer/HCC risk (according to viral infection).

		Class I			Class II				
	Type (N)	A*24:02	B*52:01	C*12:02	DRB1*15:02	DQA1*01:03	DQB1*06:01	DPA1*02:01	DPB1*09:01
		OR^a^ (95% CI) *P* value	OR^a^ (95% CI) *P* value	OR^a^ (95% CI) *P* value	OR^a^ (95% CI) *P* value	OR^a^ (95% CI) *P* value	OR^a^ (95% CI) *P* value	OR^a^ (95% CI) *P* value	OR^a^ (95% CI) *P* value
Liver cancer	Liver cancer (1,684)	**1.10** **(1.03–1.18)** **7.0E−03**	**1.26** **(1.14–1.39)** **5.8E−06**	**1.25** **(1.14–1.38)** **7.9E−06**	**1.30** **(1.18–1.4)** **3.1E−07**	**1.24** **(1.15–1.35)** **1.7E−07**	**1.27** **(1.17–1.38)** **1.3E−08**	**1.17** **(1.07–1.28)** **6.1E−04**	**1.26** **(1.13–1.40)** **2.3E−05**
	HBV (128)	0.96 (0.74**–**1.24) 7.4E−01	**1.74** **(1.27–2.38)** **5.9E−04**	**1.74** **(1.27–2.39)** **5.7E−04**	**1.95** **(1.43–2.67)** **2.8E−05**	**1.99** **(1.53–2.59)** **2.5E−07**	**2.06** **(1.58–2.67)** **6.9E−08**	**1.54** **(1.14–2.07)** **4.4E−03**	**1.73** **(1.24–2.42)** **1.4E−03**
	HCV (622)	**1.17** **(1.04–1.31)** **7.7E−03**	**1.30** **(1.11–1.53)** **1.0E−03**	**1.31** **(1.12–1.53)** **9.5E−04**	**1.29** **(1.09–1.52)** **2.9E−03**	**1.18** **(1.03–1.35)** **1.8E−02**	**1.20** **(1.05–1.37)** **9.1E−03**	1.16 (1.00**–**1.34) 5.6E−02	**1.27** **(1.07–1.51)** **6.3E−03**
	NBNC (277)	1.09 (0.92**–**1.30) 3.1E−01	1.08 (0.84**–**1.39) 5.5E−01	1.06 (0.82**–**1.37) 6.4E−01	1.17 (0.90**–**1.50) 2.4E−01	1.09 (0.89**–**1.34) 4.0E−01	1.10 (0.90**–**1.36) 3.6E−01	1.11 (0.88**–**1.39) 3.8E−01	1.22 (0.94**–**1.59) 1.3E−01
HCC	HCC (788)	1.04 (0.94**–**1.15) 4.7E−01	**1.23** **(1.07–1.42)** **4.5E−03**	**1.22** **(1.06–1.41)** **6.7E−03**	**1.32** **(1.14–1.52)** **2.1E−04**	**1.31** **(1.17–1.48)** **5.2E−06**	**1.34** **(1.19–1.50)** **1.3E−06**	**1.18** **(1.04–1.35)** **1.4E−02**	**1.26** **(1.08–1.47)** **3.2E−03**
	HBV (67)	0.89 (0.62**–**1.27) 5.3E−01	**2.13** **(1.41–3.21)** **3.0E−04**	**2.13** **(1.41–3.21)** **3.0E−04**	**2.43** **(1.62–3.63)** **1.7E−05**	**2.48** **(1.75–3.51)** **3.8E−07**	**2.55** **(1.80–3.62)** **1.5E−07**	**1.62** **(1.08–2.43)** **1.9E−02**	**2.14** **(1.39–3.30)** **5.7E−04**
	HCV (299)	1.14 (0.97**–**1.35) 1.1E−01	1.19 (0.94**–**1.51) 1.5E−01	1.18 (0.93**–**1.49) 1.8E−01	1.23 (0.97**–**1.57) 9.0E−02	**1.24** **(1.03–1.51)** **2.6E−02**	**1.27** **(1.05–1.54)** **1.4E−02**	1.06 (0.85**–**1.32) 5.9E−01	1.14 (0.88**–**1.48) 3.2E−01
	NBNC (130)	1.02 (0.79**–**1.31) 8.9E−01	1.05 (0.72**–**1.52) 8.2E−01	1.01 (0.69**–**1.48) 9.6E−01	1.19 (0.82**–**1.72) 3.6E−01	1.08 (0.80**–**1.46) 6.3E−01	1.09 (0.80**–**1.47) 6.0E−01	1.12 (0.80**–**1.55) 5.1E−01	1.27 (0.87**–**1.84) 2.2E−01

^a^Adjusted for age, sex, and the top five major PCA components, which were obtained from the pancancer GWAS.

Results with statistically significant associations are shown in bold.

HCC: hepatocellular carcinoma; HBV: hepatitis B virus; HCV: hepatitis C virus; NBNC; non-B non-C; OR: odds ratio; CI: confidence interval.

Next, ORs were calculated according to genotype. Homozygosity for the risk alleles exhibited significantly higher ORs for HBV-related liver cancer/HCC risk than heterozygosity, suggesting that risk-associated HLA alleles have synergistic effects (Table 4; the OR of homozygotes of the DRB1*15:02 allele for HBV-related HCC = 9.82, *P* = 1.2 × 10^–8^). By contrast, the ORs for A*24:02 genotypes were not significant, which is consistent with a lack of association with the HLA-A*24:02 allele (Table 3).

**Table 4:** Association of the Asian-prevalent HLA haplotype with heterozygous or homozygous retention in HBV-related liver cancer.

Liver cancer	Zygosity	Control	Cancer (Liver cancer)	OR^a^	95% CI		*P* value	Cancer (HCC)	OR^a^	95% CI		*P* value
HLA type		Number	Number		Lower	Upper		Number		Lower	Upper	
A*24:02	−/−	42,698	57	1.00				30	1.00			
	+ /−	49,678	50	0.69	0.62	1.38	6.90E−01	28	0.82	0.51	1.34	4.32E−01
	+ / +	14,615	21	1.45	0.87	2.42	1.57E−01	9	0.98	0.49	1.98	9.54E−01
B*52:01	−/−	83,864	86	1.00				43	1.00			
	+ /−	21,493	33	1.41	0.95	2.09	9.20E−02	17	1.37	0.79	2.39	2.61E−01
	+ / +	1411	7	**4.27**	**1.99**	**9.17**	**2.00E−04**	6	**7.25**	**3.13**	**16.83**	**4.00E−06**
C*12:02	−/−	83,888	88	1.00				44	1.00			
	+ /−	21,457	33	1.38	0.93	2.05	1.12E−01	17	1.35	0.78	2.34	2.90E−01
	+ / +	1,405	7	**4.20**	**1.96**	**9.03**	**2.33E−04**	6	**7.14**	**3.08**	**16.55**	**5.00E−06**
DRB1*15:02	−/−	85,751	88	1.00				44	1.00			
	+ /−	20,006	32	1.45	0.97	2.16	7.10E−02	16	1.36	0.77	2.38	2.85E−01
	+ / +	1,205	8	**5.62**	**2.74**	**11.54**	**3.00E−06**	7	**9.82**	**4.48**	**21.57**	**1.23E−08**
DQA1*01:03	−/−	70,892	62	1.00				30	1.00			
	+ /−	32,360	51	**1.53**	**1.08**	**2.18**	**1.80E−02**	25	1.38	0.84	2.26	2.06E−01
	+ / +	3,845	15	**3.52**	**2.05**	**6.04**	**5.00E−06**	12	**5.78**	**3.09**	**10.80**	**3.96E−08**
DQB1*06:01	−/−	71,705	62	1.00				30	1.00			
	+ /−	31,697	51	**1.58**	**1.11**	**2.25**	**1.20E−02**	25	1.42	0.87	2.33	1.66E−01
	+ / +	3,663	15	**3.69**	**2.15**	**6.34**	**2.00E−06**	12	**6.06**	**3.24**	**11.33**	**1.67E−08**
DPA1*02:01	−/−	77,361	82	1.00				43	1.00			
	+ /−	27,270	37	1.20	0.82	1.75	3.62E−01	18	1.08	0.63	1.85	7.81E−01
	+ / +	2,469	9	**3.17**	**1.61**	**6.25**	**8.82E−04**	6	**4.11**	**1.78**	**9.53**	**9.67E−04**
DPB1*09:01	−/−	87,385	94	1.00				47	1.00			
	+ /−	18,539	28	1.34	0.88	2.04	1.74E−01	15	1.38	0.78	2.45	2.75E−01
	+ / +	1,028	6	**4.97**	**2.18**	**11.32**	**1.34E−04**	5	**8.16**	**3.27**	**20.36**	**7.00E−06**
Asian haplotype^b^	−/−	90,093	99	1.00				48	1.00			
	+ /−	12,319	15	1.04	0.60	1.78	8.97E−01	9	1.21	0.60	2.45	5.93E−01
	+ / +	672	5	**6.44**	**2.62**	**15.82**	**5.00E−05**	4	**10.10**	**3.66**	**27.89**	**8.00E−06**

^a^Adjusted for age, sex, and the top five major PCA components, which were obtained from the pancancer GWAS.

Results with statistically significant associations are shown in bold.

HCC: hepatocellular carcinoma; HBV: hepatitis B virus; HCV: hepatitis C virus; NBNC; non-B non-C; OR: odds ratio; CI: confidence interval.

^b^Includes B*52:01, C*12:02, DRB1*15:02, DQA1*01:03, DQB1*06:01, DPA1*02:01, and DPB1*09:01.

### Affinity of HLA-class II molecules for HBV-derived peptides

**Figure 1:**
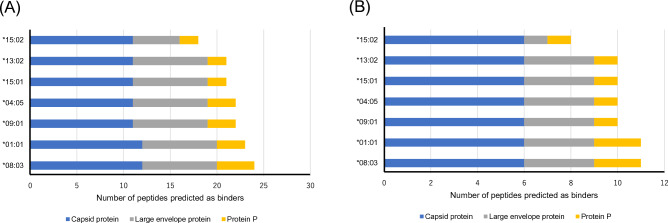
Potential inefficient binding of the DRB1*15:02 molecule. Peptides derived from HBV that were predicted to bind with high affinity (i.e., predicted score > 0.95) to HBV peptides cataloged in the IEDB were identified by the MARIA program. The results for all deposited peptides (left panel, n = 1551) and for peptides that elicited a reaction in a T cell assay (right panel, n = 465) are shown. Colors indicate the protein type from which the binder peptides are derived.

The association between HLA-class II alleles and HBV-related liver cancer prompted us to investigate whether the HLA-class II molecules encoded by the risk-associated alleles bind efficiently to HBV-derived peptides since HLA-class II molecules play a pivotal role in immune responses to viral infection^19,20^. Using the antigen-prediction algorithm MARIA^21^, we estimated the fraction of peptides efficiently captured by polymorphic HLA-DRB1 proteins among 1551 HBV-derived peptides deposited in the IEDB. Interestingly, we found that the DRB1*15:02 molecule bound fewer neoantigens than other HLA-DRB1 molecules (Fig. 1).

### Immune profile of HBV-related HCC in risk allele carriers

**Figure 2:**
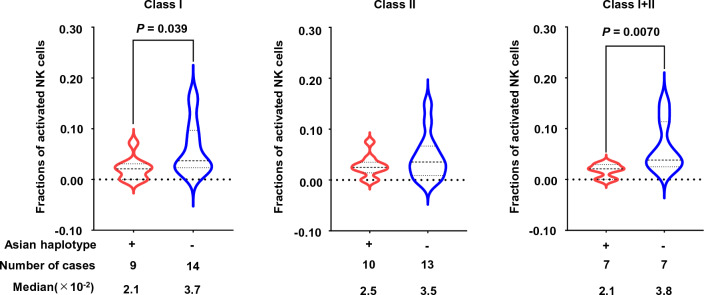
Proportion of activated NK cells infiltrating HCC tissues. Fractions of activated NK cells within HCC tissues was determined using the CIBERSORTx algorithm using RNA sequencing data obtained from 160 Japanese patients with HCC. Fractions of activated NK cells was stratified according to the HLA-class I, -class II, and -class I/II genotypes. Statistical significance was determined by the Mann–Whitney U test, and *P* values < 0.05 were considered significant.

Often, HBV-associated HCCs exhibit reduced intratumoral infiltration by activated NK cells^22^, which represents an immune profile that could potentially promote tumor development and progression^23^. Therefore, to investigate whether the risk-associated Asian-prevalent HLA haplotype plays a role in this phenotype, we analyzed RNA sequencing and whole-exome sequencing data from 160 Japanese HCC samples^24^. HCC cases with the HLA-class I and -class II haplotypes were identified from noncancerous tissue whole-exome sequencing data, while intratumoral immune cell fractions were estimated from tumor tissue RNA sequencing data using the CIBERSORTx program1^25^. Consistent with a previous report^22^, we found that the fraction of activated NK cells in HBV-related HCCs was lower than that in NBNC-HCCs (Supplementary Fig. 1), although the difference was not statistically significant (*P* > 0.05; Mann–Whitney U test). HCCs with the Asian-prevalent HLA haplotype had a lower percentage of activated NK cells than those without (Fig. 2), suggesting that intratumor infiltration by activated NK cells is lower in carriers of the HCC risk allele. By contrast, there was no significant difference in intratumor infiltration by CD8+, CD4+, and dendritic cells (*P* > 0.05; Mann–Whitney U test; Supplementary Fig. 2).

## Discussion

Here, we conducted a comprehensive association analysis of HLA alleles to determine their role in pancancer risk and in the risk of developing 12 specific cancer types. The results clearly indicate that HLA-class I and -class II alleles comprising an Asian-prevalent haplotype play a role in the risk of developing Asian-prevalent cancers such as liver, cervical, and stomach cancers. To the best of our knowledge, this is the first investigation that has provided evidence that HLA polymorphisms are an endogenous factor contributing to the risk of Asian-prevalent cancers. Of the 12 cancer types, we focused on liver cancer because it is prevalent in Asia, and information regarding viral infections is readily available. Remarkably, HLA alleles comprising the Asian-prevalent haplotype showed a more pronounced association with the risk of HBV-related HCC than HCV-related and NBNC-HCCs, a finding in line with the high incidence rate of HBV-related HCC in Asian countries, including Japan^26,27^. These HLA alleles did not deviate significantly from Hardy–Weinberg equilibrium (Supplementary Table 4), and showed linkage disequilibrium (Supplementary Fig. 3) in our study population. Therefore, these alleles, and the haplotype, are a common genetic risk factor shared by the Japanese population.

Two particular findings provide insight into the molecular mechanisms underlying the way in which HLA polymorphisms contribute to the risk of HBV-related HCC. First, in silico deductions suggest that the HLA-DRB1*15:02 molecule encoded by the Asian haplotype does not bind HBV-derived peptides efficiently. In particular, inefficient binding of the HLA-DRB1*15:02 molecule to large envelope protein-derived peptides is plausible, as these proteins comprise the hepatitis B surface antigen (HBsAg). We then used whole-exome sequencing data from 160 Japanese HCC cases to investigate the ability of HLA-class I molecules, specifically those encoded by alleles associated with increased risk (i.e., B*52:01 and C*12:02), to bind efficiently to neoantigens arising from somatic mutations in tumor tissues. We deduced that the number of neoantigens that bound to these HLA-class I molecules was not lower than the number that bound to other HLA-class I molecules (Supplementary Fig. 4A). Notably, a previous study showed that HLA-class II alleles, but not class I alleles, comprising the Asian-prevalent haplotype are associated with an increased risk for chronic hepatitis B infection in Japanese individuals^20^. As such, it is possible that this haplotype contributes to the risk of liver cancer by increasing the likelihood of developing a chronic hepatitis B infection, rather than by promoting evasion of immune surveillance by established tumor cells.

The transcriptome data from the 160 HCCs enabled us to investigate the impact of the haplotype on the immunogenic properties of HCC. Chronic HBV infection, the primary risk factor for HCC, modulates expression of inhibitory and activating receptors on NK cells within tumor tissues, leading to a decrease in NK cell activation^23^. In fact, HBV-associated HCCs frequently exhibit reduced infiltration by activated NK cells^22^, which is an observation made in the present study. Moreover, HCCs carrying the risk haplotype had a lower fraction of activated NK cells than those without the haplotype. The mechanisms underlying these observations remain unclear. Previous studies suggest that HLA-class I molecules expressed on tumor cells mediate NK cell suppression^28^. Intriguingly, we observed infrequent LOH of the B*52:01 and C*12:02 alleles (which are associated with increased risk) compared with other class I alleles in HCCs of individuals heterozygous for these alleles (Supplementary Fig. 4B). This suggests that retention of these HLA-class I molecules may contribute to NK cell suppression. Nevertheless, further functional investigations are needed to establish a definitive conclusion.

In summary, our investigation highlights that the Asian-prevalent haplotype encompassing HLA-class I and -class II alleles increases susceptibility to Asian-prevalent malignancies, specifically HBV-related HCC, by suppressing antiviral and antitumor immune responses. Nonetheless, certain limitations must be acknowledged. First, although our study was a large-scale association analysis encompassing 12 major cancer types, the sample size for each cancer type, including HBV-related HCC, was small. Additionally, only Japanese patients were included in our analysis; thus, the associations should be confirmed in larger and more diverse cohorts of HCC patients. Second, the transcriptome analysis of HCC focusing on NK cells was performed using only publicly available data, thereby limiting our ability to assess detailed pathological characteristics such as immune cell distribution. Therefore, the relationship between HLA alleles and the intratumoral distribution of immune cells should be evaluated further using immunohistochemical methods coupled with HLA genotype data.

## Materials and methods

### Patients characteristics

Details of the BBJ subjects, including liver cancer cases, were described previously^29^. The BBJ project enrolled participants, including cases diagnosed as liver cancer, from healthcare facilities in Japan; history of HBV or HCV infection (yes, no, or unknown) was obtained from medical records, and from interviews using a standardized questionnaire at enrolment. The histological type of liver cancer was diagnosed on the basis of tissue or cytological samples obtained from biopsies. Liver cancer histology was not available for a subset of the patients because current guidelines for liver cancer from the Japan Society of Hepatology indicate that a diagnosis of HCC is sometimes based only on imaging results.

### Subjects enrolled in the association analysis of HLA alleles

Genome-wide genotype data from 174,696 individuals were analyzed for the presence of 55,225 SNPs in the MHC region. The data were obtained from blood DNA samples collected through genome-wide SNP chip analysis using Illumina Human OmniExpress v1, Human OmniExpressExome v1.0, or Human OmniExpressExome v1.2 BeadChips. The data were obtained from the BioBank Japan (BBJ) database (approval number: P0067 and P0078), which is a national project that began in 2003 to collect DNA and clinical information from a total of 200,000 patients with at least one of 47 common diseases, including 12 types of cancer (http://biobankjp.org). The 174,696 subjects included 33,471 cancer cases and 141,225 controls. Principal component analysis of the 55,225 SNPs from the BBJ subjects was performed using 1,000 Genomes Project SNP data from Japanese (n = 91), Chinese (n = 190), European (n = 280), and African populations (n = 550)^8^. This enabled construction of a study population comprising 138,830 individuals with matched genetic backgrounds, including 31,727 cancer cases and 107,103 noncancer controls (Supplementary Fig. 5 and Supplementary Table 1).

### HLA genotyping and association analysis

SNP2HLA^30^ and a Japanese HLA reference panel^8^ were used to impute the HLA alleles of the study subjects from genotype data covering the MHC region. This enabled identification of four-digit classical class I (HLA-A, B, and C) and class II (HLA-DRB1, DQA1, DQB1, DPA1, and DPB1) HLA alleles. Chi-squared tests and logistic association analyses of case/control data were performed for each HLA allele, and the 12 cancer types, using the PLINK program (version 1.07)^31^; age, sex, and the top five principal component scores were used as covariates. Bonferroni correction was applied to account for multiple testing. A total of 147 HLA-class I/II alleles were studied. *P* < 3.4 × 10^–4^ was considered significant (i.e., *P* < 3.4 × 10^–4^ = 0.05/147). Statistical analyses were performed using R statistical environment version 4.1.2 (GraphPad Prism9, or PLINK1.07).

### In silico prediction of the binding of polymorphic HLA-DRB1 molecules to HBV peptides

The amino acid sequences of 1551 peptides derived from HBV were obtained from the Immune Epitope Database (IEDB)^32^. These peptides comprised fragments of proteins encoded by the HBV genome, including the capsid protein (n = 334), external core antigen (n = 202), large envelope protein (n = 555), protein P (n = 317), and protein X (n = 143). The MARIA (https://maria.stanford.edu/index.php) program, which specifically predicts peptide binding to HLA-DRB1 molecules, was used to infer the potential binding ability of these peptides to polymorphic HLA-DRB1 molecules. Peptides with a predicted score > 0.95 were considered to have positive binding ability, consistent with previous studies^21^.

### Computational identification of HLA-class I molecule-restricted neoantigens

Whole-exome sequencing data (Fastq files) from HCC and nontumor tissue DNA samples obtained from 160 HCC patients of Japanese descent were procured from the National Bioscience Database Center (NBDC) Human Database (research ID: hum0187.v2)^24^. Exome sequencing was conducted on the Illumina HiSeq2000 platform using 2 × 100 bp paired-end reads (resulting in an estimated 100-fold coverage) and the SureSelect Human All Exon Kit V4/V5 (Agilent Technologies, Santa Clara, United States) or a SeqCap EZ HGSC VCRome2.1 design1 kit (Roche, Basel, Switzerland). Basic alignment and sequence quality control were then undertaken in accordance with the GATK4 best practices pipeline^33^. The reads obtained were aligned to the UCSC human genome 38 (hg38) reference sequence. Somatic single nucleotide variants and insertion/deletion variants were detected by the Mutect2 program (BROAD Institute; http://www.broadinstitute.org/gatk/). Each nonsynonymous single nucleotide variant was translated into a 17-mer peptide sequence centered on the mutated amino acid. The 17-mers were then used to generate 9-mers through a sliding window approach, followed by prediction of HLA-class I binding to neopeptides by the HLAthena program^34^. Neoantigens were selected based on a prediction score of Msi > 0.9 for each patient-specific HLA-class I allele^35–38^.

### Estimation of somatic HLA-class I allele loss in HCC

The HLA-class I genotypes (comprising four-digit alleles) of the HCC patients were determined from whole-exome sequencing data using the HLA-HD^39^ and POLYSOLVER^40^ programs. Somatic loss of HLA-class I alleles was estimated by the LOHHLA^41^ program using default settings. In short, the allele-specific copy number of each HLA-class I locus was determined by realigning sequence reads to patient-specific HLA reference sequences. Somatic loss of heterozygosity (LOH) was considered positive when the difference in the log copy ratio between the two HLA alleles was less than the Pval_unique value of 0.01, as previously described^42^.

### RNA sequencing and immune cell profiling

The RNA sequencing data (Fastq files) for tumor tissues from the same cohort of 160 HCC patients were acquired from the NBDC (research ID: hum0187.v2). The polyadenylated RNA libraries were synthesized using the TruSeq Stranded mRNA Library Prep kit (Illumina) and sequenced using the Illumina HiSeq2000 platform, generating 2 × 100 bp paired-end reads. Read alignment was performed using STAR version 2.7.3a^43^, with the human genome (GRCh38) and transcriptome data (GENCODE version 31^44^) as reference datasets. Transcripts per million (TPM) values were calculated using the StringTie program (version 2.0.4)^45^. Levels of immune infiltration were calculated from TPM expression data using the LM22 gene signature and the CIBERSORTx algorithm^25,46^. Data were run with 1000 permutations under the LM22 signature. The fraction of CD4+ cells was calculated by summing the fractions of “T cells CD4 naïve”, “T cells CD4 memory resting”, and “T cells CD4 memory activated”. The fraction of dendritic cells was calculated by summing the fractions of “dendritic cells resting” and “dendritic cells activated”.

### Statistical analysis

Logistic regression analysis of HLA alleles/haplotypes was conducted using the PLINK program (version 1.07), with age, sex, and the top five principal component scores (described in “Subjects enrolled in the association analysis of HLA alleles”) as covariates. The significance of differences in the distribution of values, such as the number of neoantigens between two groups, was assessed using the Mann–Whitney U test. The binomial distribution test was used to evaluate the presence of LOH in tumors. All statistical analyses were performed using SPSS Statistics version 27 (IBM, Tokyo, Japan), the R statistical environment version 4.1.2, GraphPad Prism 9.0 (Dotmatics, Boston, United States), or PLINK 1.07.

### Supplementary Information

Supplementary Tables.

Supplementary Figures.

## Data Availability

The genotype and phenotype data of BBJ subjects can be accessed through procedures described on its website (research ID: hum0014.v6.158k.v1 and hum0028.v2) (https://humandbs.biosciencedbc.jp/en/hum0014-v21 and https://humandbs.biosciencedbc.jp/en/hum0028-v2). The transcriptome and whole exome data of liver cancer used in this study are publicly available at the NBDC Human Database (research ID: hum0187.v2) (https://humandbs.biosciencedbc.jp/en/hum0187-v2). Peptide sequences of HBV are obtained from public databases IEBD (http://iedb.org) without restriction.
