# Supplementary material for: Contribution of an Asian-prevalent HLA haplotype to the risk of HBV-related hepatocellular carcinoma
Source: Sci Rep. 2023 Aug 9;13:12944. doi: 10.1038/s41598-023-40000-3 (PMC10412552; doi:10.1038/s41598-023-40000-3)
Supplement: Supplementary file 1 — Supplementary Tables. [file 41598_2023_40000_MOESM1_ESM.docx]

| **Supplementary Table 1.** Cancer cases and non-cancer controls used for the association study | | |
| --- | --- | --- |
| Subject | Type of cancer | N |
| Case |  | 31727^a^ |
|  | Colon cancer | 6,854 |
|  | Stomach cancer | 6,424 |
|  | Breast cancer | 5,476 |
|  | Prostate cancer | 5,311 |
|  | Lung cancer | 3,919 |
|  | Liver cancer | 1,684 |
|  | Esophageal cancer | 1,274 |
|  | Uterine cancer | 975 |
|  | Ovarian cancer | 704 |
|  | Cervical cancer | 523 |
|  | Pancreatic cancer | 408 |
|  | Gallbladder & bile duct cancer | 318 |
| Control* |  | 107,103 |
| *Control cases included cerebral infarction, epilepsy, asthma, ischemic heart disease, arrhythmia, heart failure, chronic hepatitis B/C, liver cirrhosis, osteoporosis, diabetes mellitus, dyslipidemia, rheumatoid arthritis, atopic dermatitis, glaucoma, cataract, and periodontal disease. | | |
|  |  |  |
|  |  |  |
|  |  |  |
| ^a^ This number includes 2,143 cases with multiple primary tumors. | | |

| **Supplementary Table 2.** Top 10 most common haplotypes in the Japanese population | | | | | | | | | |
| --- | --- | --- | --- | --- | --- | --- | --- | --- | --- |
| **Ranking** | **A** | **C** | **B** | **DRB1** | **DQA1** | **DQB1** | **DPA1** | **DPB1** | **Haplotype**  **frequency (%)^a^** |
| 1 | ***24:02** | ***12:02** | ***52:01** | ***15:02** | ***01:03** | ***06:01** | ***02:01** | ***09:01** | 7.32 |
| 2 | ***24:02** | *07:02 | *07:02 | *01:01 | *01:01 | *05:01 | *01:03 | *04:02 | 3.19 |
| 3 | *33:03 | *14:03 | *44:03 | *13:02 | *01:02 | *06:04 | *01:03 | *04:01 | 3.13 |
| 4 | ***24:02** | *01:02 | *54:01 | *04:05 | *03:03 | *04:01 | *02:02 | *05:01 | 1.99 |
| 5 | *11:01 | *04:01 | *15:01 | *04:06 | *03:01 | *03:02 | *01:03 | *02:01 | 1.21 |
| 6 | *02:07 | *01:02 | *46:01 | *08:03 | ***01:03** | ***06:01** | *02:02 | *02:02 | 0.92 |
| 7 | ***24:02** | ***12:02** | ***52:01** | ***15:02** | ***01:03** | ***06:01** | *02:02 | *05:01 | 0.79 |
| 8 | *02:07 | *01:02 | *46:01 | *08:03 | ***01:03** | ***06:01** | *02:02 | *05:01 | 0.70 |
| 9 | *11:01 | *01:02 | *54:01 | *04:05 | *03:03 | *04:01 | *02:02 | *05:01 | 0.66 |
| 10 | ***24:02** | *01:02 | *59:01 | *04:05 | *03:03 | *04:01 | *01:03 | *04:02 | 0.53 |
| ^a^Data were obtained from the HLA Laboratory (<https://hla.or.jp/med/frequency_search/en/haplo/>). Eight HLA alleles associated with risk are in bold. | | | | | | | | | |

| **Supplementary Table 3.** Analysis of the association of HLA alleles with HBV- and HCV-related liver cancer/HCC risk (case-case analysis) | | | | | | | | | |
| --- | --- | --- | --- | --- | --- | --- | --- | --- | --- |
|  |  | Class Ⅰ | | | Class Ⅱ | | | | |
|  | Type  (N) | A*24:02 | B*52:01 | C*12:02 | DRB1*15:02 | DQA1*01:03 | DQB1*06:01 | DPA1*02:01 | DPB1*09:01 |
|  |  | OR^a^ (95% CI) *P* value | OR^a^ (95% CI) *P* value | OR^a^ (95% CI) *P* value | OR^a^ (95% CI) *P* value | OR^a^ (95% CI) *P* value | OR^a^ (95% CI) *P* value | OR^a^ (95% CI) *P* value | OR^a^ (95% CI) *P* value |
|  |  |  |  |  |  |  |  |  |  |
| Liver cancer | HBV  (128) | 0.89 (0.64**—**1.24) 5.0E-01 | **1.66 (1.07—2.58) 2.4E-02** | **1.67 (1.08—2.59) 2.3E-02** | **1.61 (1.04—2.50) 3.4E-02** | **1.76 (1.21—2.54) 2.9E-03** | **1.80 (1.24—2.61) 1.9E-03** | 1.21 (0.81**—**1.83) 3.5E-01 | 1.40 (0.88**—**2.25) 1.6E-01 |
|  | HCV  (622) | 1.07 (0.87**—**1.31) 5.4E-01 | 1.19 (0.88**—**1.61) 2.6E-01 | 1.21 (0.89**—**1.64) 2.2E-01 | 1.09 (0.80**—**1.48) 5.8E-01 | 1.07 (0.84**—**1.37) 5.8E-01 | 1.08 (0.85**—**1.38) 5.4E-01 | 1.05 (0.80**—**1.37) 7.3E-01 | 1.04 (0.75**—**1.42) 8.2E-01 |
| HCC | HBV  (67) | 1.07 (0.65**—**1.75) 8.0E-01 | **2.11 (1.13—3.97) 2.0E-02** | **2.13 (1.14—4.00) 1.9E-02** | **2.16 (1.14—4.09) 1.8E-02** | **2.36 (1.36—4.11) 2.3E-03** | **2.47 (1.42—4.31) 1.4E-03** | 1.47 (0.81**—**2.66) 2.0E-01 | **2.01 (1.00—4.01) 4.9E-02** |
|  | HCV  (299) | 1.10 (0.82**—**1.49) 5.2E-01 | 1.09 (0.70**—**1.71) 7.0E-01 | 1.11 (0.71**—**1.75) 6.4E-01 | 0.99 (0.63**—**1.55) 9.7E-01 | 1.16 (0.80**—**1.67) 4.3E-01 | 1.18 (0.82**—**1.70) 3.9E-01 | 0.94 (0.64**—**1.40) 7.7E-01 | 0.86 (0.54**—**1.38) 5.4E-01 |
| ^a^Adjusted for age, sex, and the top five major PCA components, which were obtained from the pan-cancer genome-wide association study. Odds ratios against NBNC cases were calculated. | | | | | | | | | |
| HLA: human leukocyte antigen; OR: odds ratio; CI: confidence interval; HCC: hepatocellular carcinoma; HBV: hepatitis B virus, HCV: hepatitis C virus. | | | | | | | | | |
| Bold text: significantly different. | | | | | | | | | |

| **Supplementary Table 4.** Hardy-Weinberg equilibrium of eight risk-associated HLA alleles | | | | | | | | | | | |
| --- | --- | --- | --- | --- | --- | --- | --- | --- | --- | --- | --- |
|  | | | Class I^a^ | | |  | Class II^a^ | | | | |
|  |  |  | A*24:02 | B*52:01 | C*12:02 |  | DRB1*15:02 | DQA1*01:03 | DQB1*06:01 | DPA1*02:01 | DPB1*09:01 |
| Case | Pan-cancer | | 0.08185 | 0.02647 | 0.0282 |  | 0.01833 | 0.1119 | 0.1211 | 0.4007 | 0.3091 |
|  | Liver cancer | | 0.4741 | 0.2237 | 0.1874 |  | 0.2038 | 0.02973 | 0.06632 | 0.8642 | 0.5599 |
|  | HCC | HCC | 0.7619 | 0.2291 | 0.2242 |  | 0.4426 | 0.2326 | 0.423 | 0.5352 | 1 |
|  |  | HBV | 0.5895 | 0.06241 | 0.06241 |  | 0.01387 | 0.1169 | 0.1169 | 0.07563 | 0.03986 |
|  |  | HCV | 1 | 1 | 1 |  | 1 | 1 | 0.8719 | 0.5189 | 0.7674 |
|  |  | NBNC | 0.1408 | 0.1302 | 0.108 |  | 1 | 0.3012 | 0.29 | 0.7526 | 0.2148 |
| Control | | | 0.4119 | 0.3958 | 0.4122 |  | 0.3282 | 0.04338 | 0.02749 | 0.2274 | 0.1996 |
| ^a^P-values for Hardy-Weinberg equilibrium are shown. None of the eight HLA alleles in case and control populations deviated significantly from Hardy-Weinberg equilibrium (i.e., P > 8.9×10^-4^=0.05/56 tests).  HCC: hepatocellular carcinoma; HBV: hepatitis B virus; HCV: hepatitis C virus; NBNC; non-B non-C. | | | | | | | | | | | |
