# Supplementary material for: Contribution of an Asian-prevalent HLA haplotype to the risk of HBV-related hepatocellular carcinoma
Source: Sci Rep. 2023 Aug 9;13:12944. doi: 10.1038/s41598-023-40000-3 (PMC10412552; doi:10.1038/s41598-023-40000-3)
Supplement: Supplementary file 2 — Supplementary Figures. [file 41598_2023_40000_MOESM2_ESM.pdf]

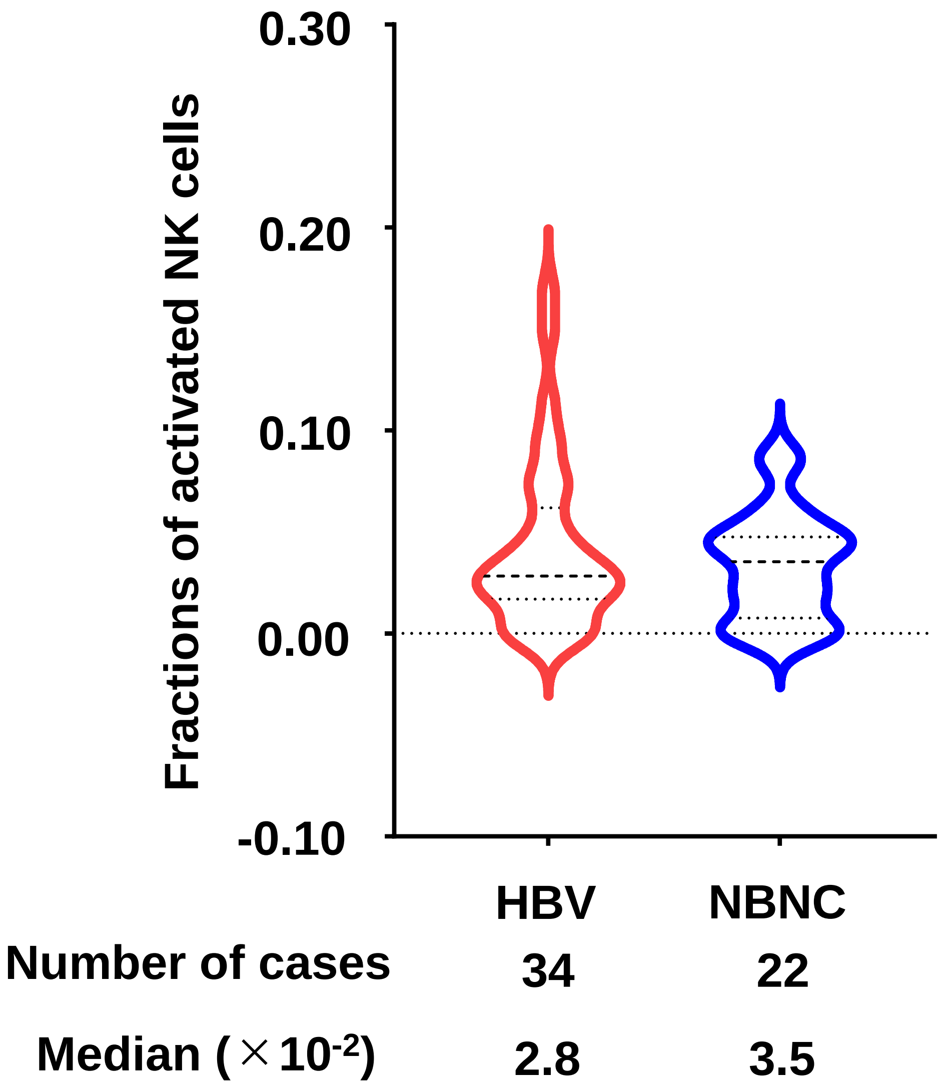

Supplementary Fig. 1 Proportion of activated NK cells infiltrating HCC tissues

Fractions of activated intratumoral NK cells was determined by analyzing RNA sequencing data from 160 HCC tissues using the CIBERSORTx algorithm. Fractions of activated NK cells within HCCs that were associated with HBV infection, and within HCCs not associated with HBV infection (NBNC-HCCs), are depicted in the figure.

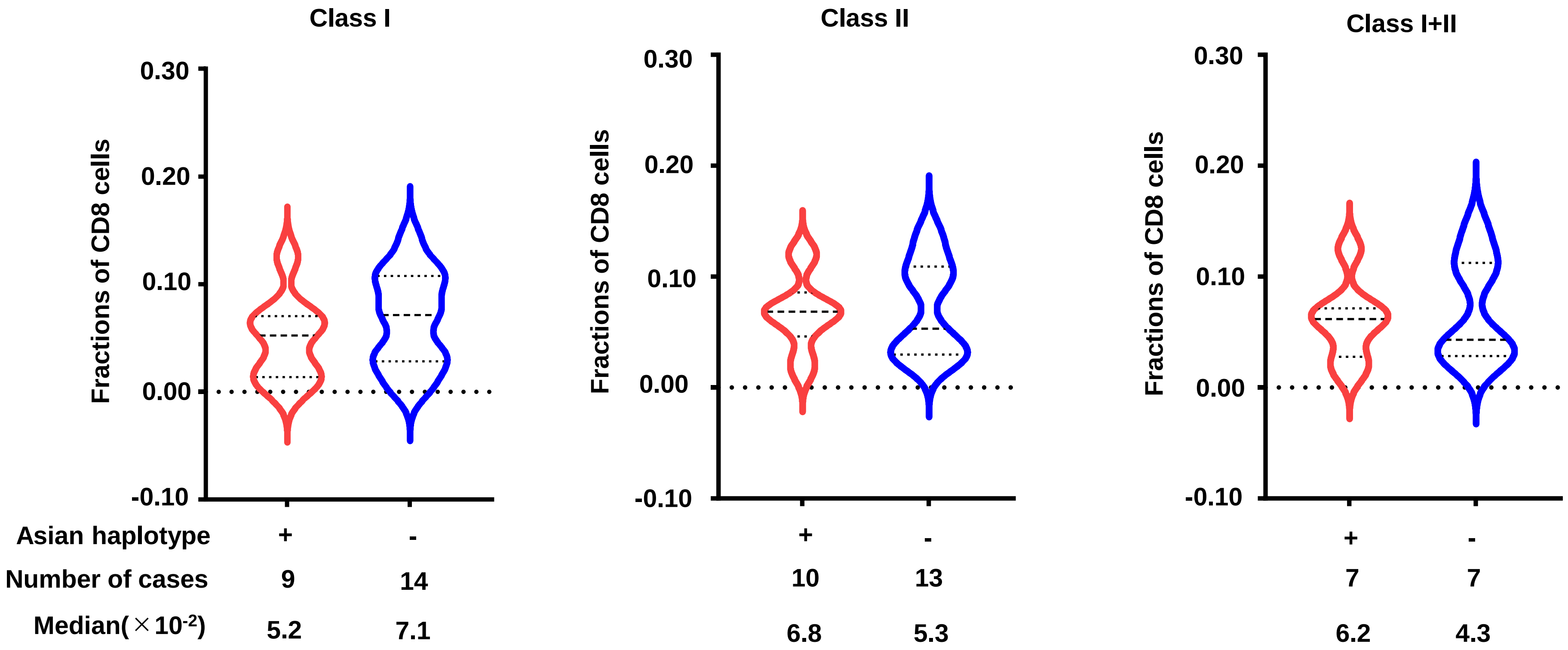

Supplementary Fig. 2A Proportion of CD8 cells infiltrating HCC tissues

Fractions of CD8 cells within HCC tissues was determined using the CIBERSORTx algorithm, based on RNA sequencing data obtained from 160 Japanese patients with HCC. The proportion was stratified according to the HLA class I, class II, and class I/II genotypes. Statistical significance was determined by the Mann–Whitney U test, and p-values < 0.05 were considered significant.

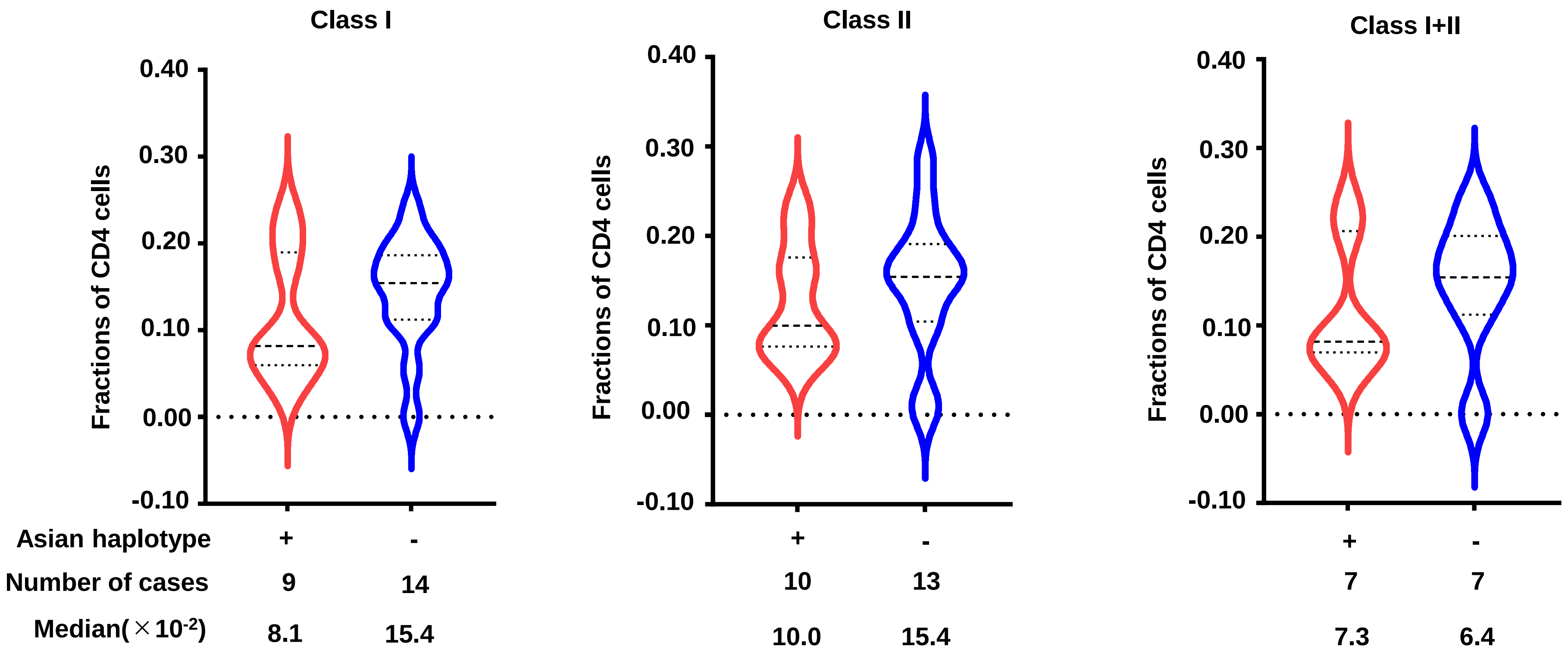

Supplementary Fig. 2B Proportion of CD4 cells infiltrating HCC tissues

Fractions of CD4 cells within HCC tissues was determined using the CIBERSORTx algorithm, based on RNA sequencing data obtained from 160 Japanese patients with HCC. The proportion was stratified according to the HLA class I, class II, and class I/II genotypes. Statistical significance was determined by the Mann–Whitney U test, and p-values < 0.05 were considered significant.

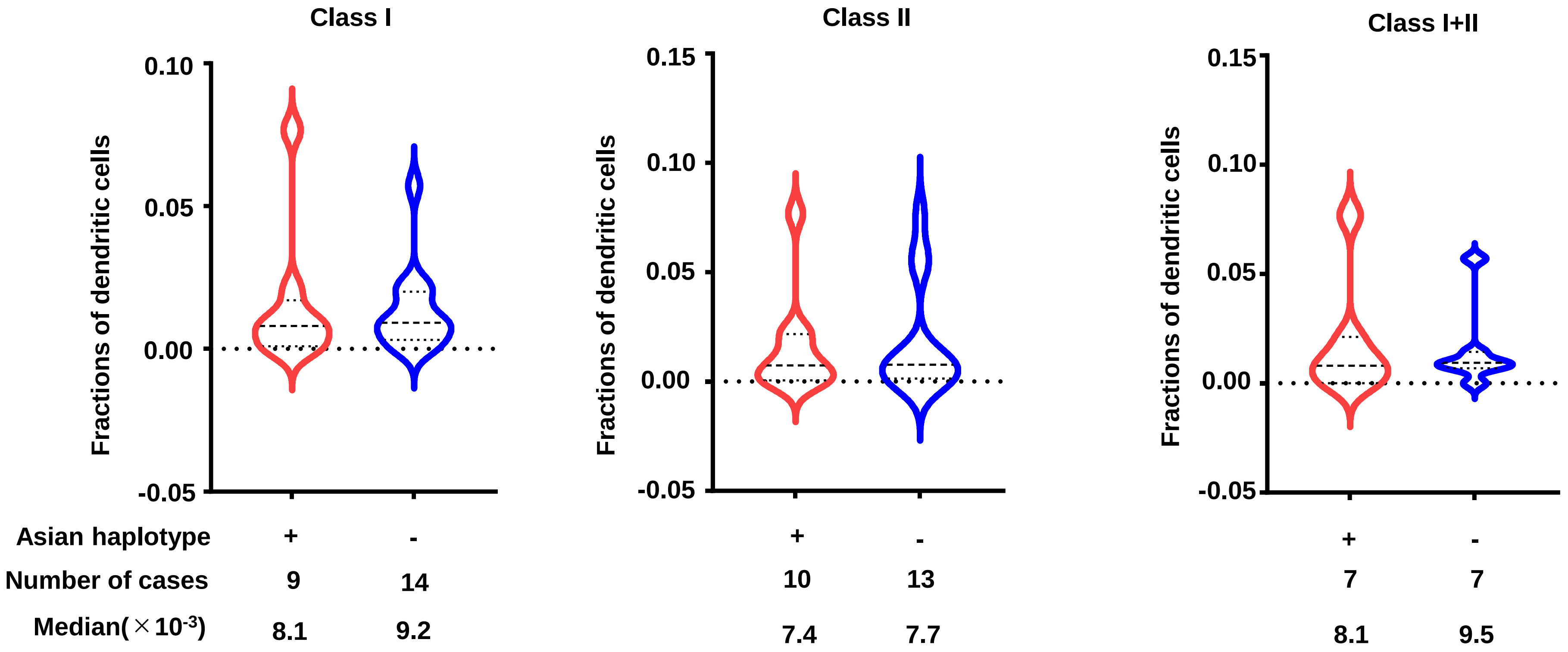

Supplementary Fig. 2C Proportion of dendritic cells infiltrating HCC tissues

Fractions of dendritic cells within HCC tissues was determined using the CIBERSORTx algorithm, based on RNA sequencing data obtained from 160 Japanese patients with HCC. The proportion was stratified according to the HLA class I, class II, and class I/II genotypes. Statistical significance was determined by the Mann–Whitney U test, and p-values < 0.05 were considered significant.

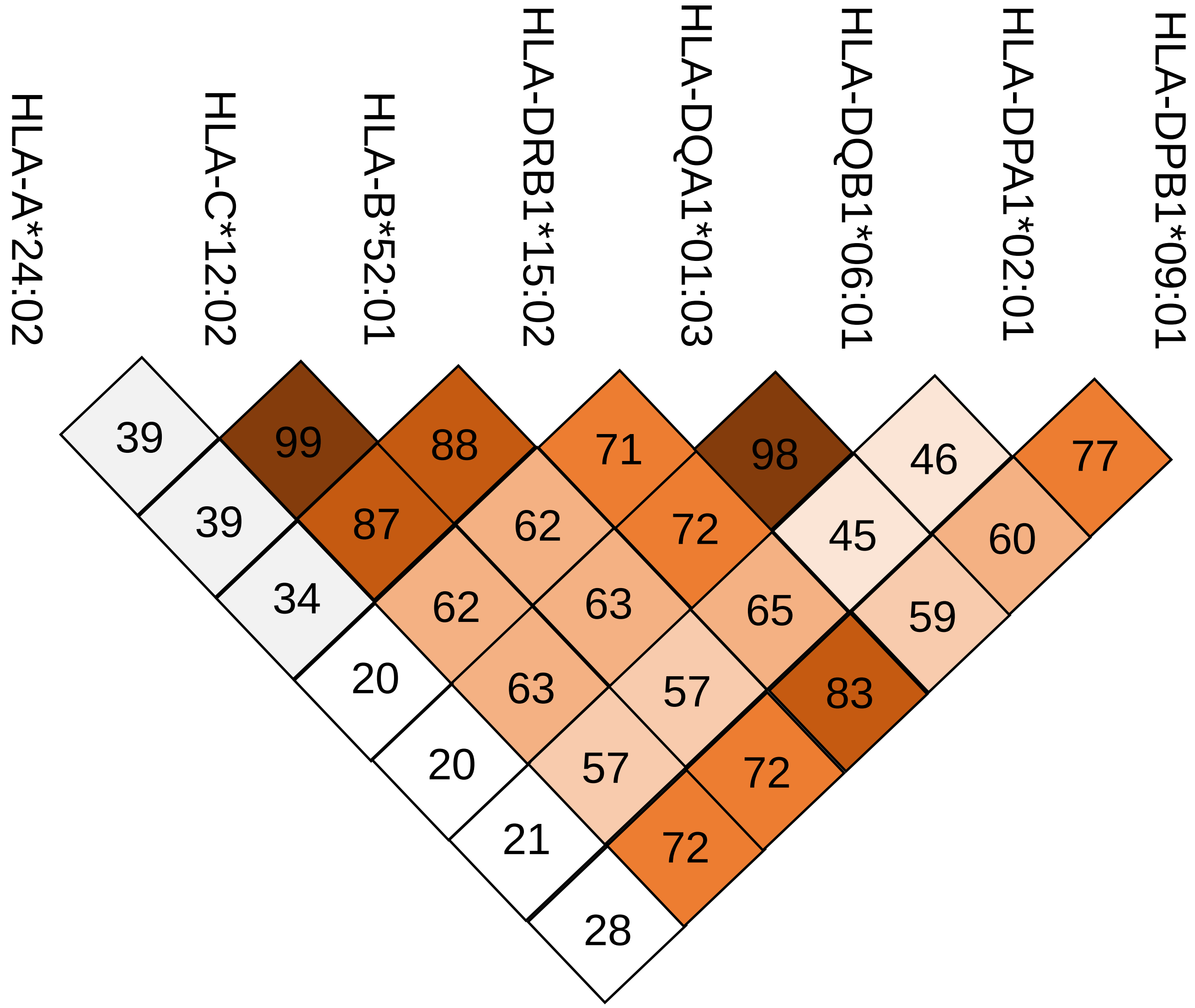

Supplementary Fig. 3 Estimation of linkage disequilibrium in HCC patients

LD plots were constructed for eight HLA alleles comprising the Asian-prevalent haplotype, with D’ values calculated by the plink program.

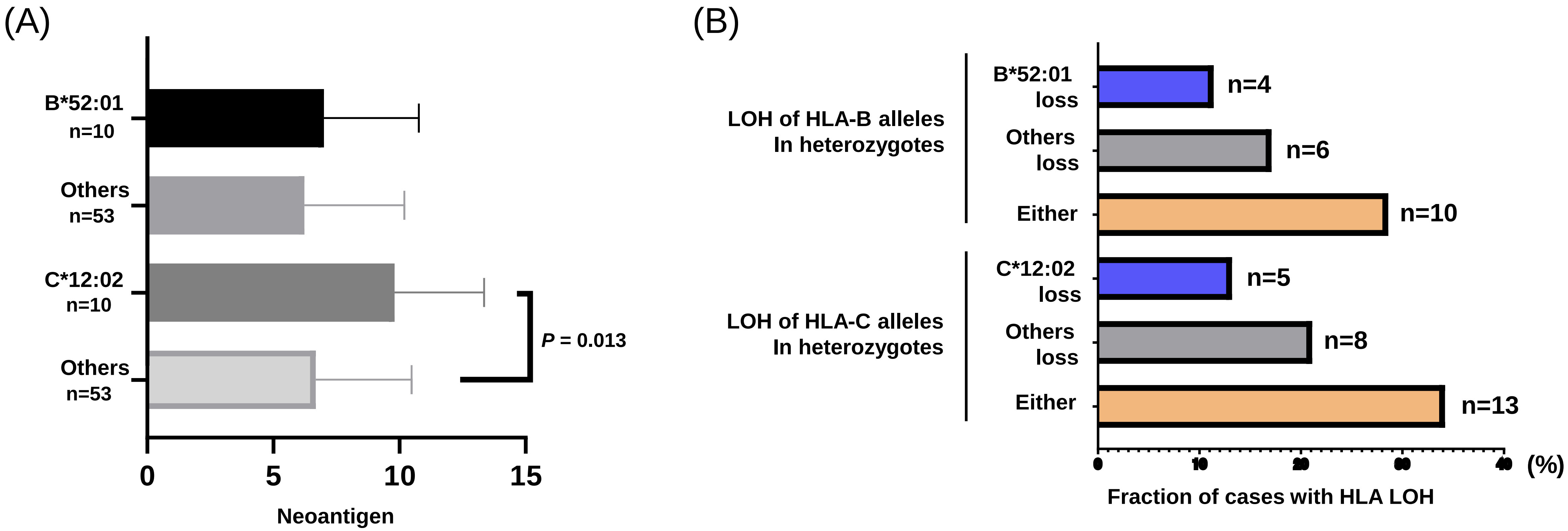

Supplementary Fig. 4 Risk-associated HLA-class I alleles and antitumor immunity

(A) The number of somatic mutation-derived peptides predicted to bind (i.e., predicted score > 0.90) to heterozygotes of B*52:01 or C*12:02 is presented. The number of peptides binding to B*52:01/C*12:02 molecules and non-B*52:01/C*12:02 allele-derived molecules in tumor specimens is also displayed. P-values, determined by the Mann–Whitney U test, are shown.

(B) Loss of HLA-class I B*52:01 or C*12:02B*52:01 or C*12:02 alleles from HCC. Loss of heterozygosity of the risk associated class I alleles, B*52:01 and C*12:02, is less than for other alleles.

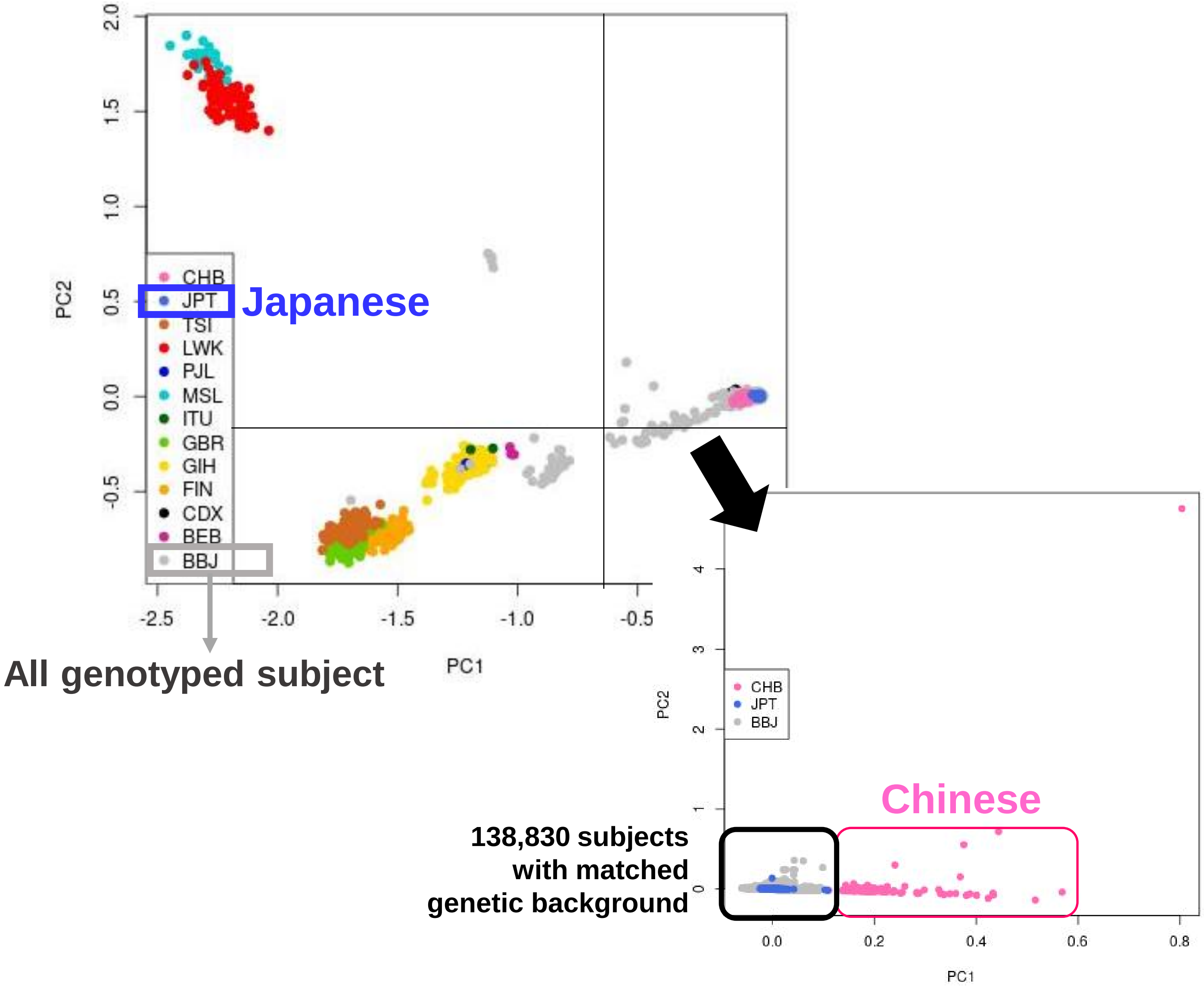

Supplementary Fig. 5 Selection of study subjects matched according to genetic background
Based on the results of principal component analysis using 55,225 SNPs, 138,830 study participants were selected from a total of 174,696 individuals. The selected participants exhibited similar PCA component values to those of 91 Japanese individuals in the 1000 Genomes Project, thereby ensuring a genetically-matched study cohort.
